# Implementation of Vital Signs Detection Algorithm for Supervising the Evacuation of Individuals with Special Needs

**DOI:** 10.3390/s25237391

**Published:** 2025-12-04

**Authors:** Krzysztof Konopko, Dariusz Janczak, Wojciech Walendziuk

**Affiliations:** Faculty of Electrical Engineering, Bialystok University of Technology, 15-351 Bialystok, Poland

**Keywords:** vital sign detection, accelerometer, pulse detection, heart rate measurement, monitoring band, smart band, evacuation, Fast Fourier Transform, kernel density estimation, IMU, PPG sensor

## Abstract

The article describes a system for monitoring the vital parameters of evacuated individuals, integrating three key functionalities: pulse detection, verification of wristband contact with the skin, and motion recognition. For pulse detection, the system employs the MAX30102 optical sensor and a signal processing algorithm presented in the study. The algorithm is based on spectral analysis using the Fast Fourier Transform (FFT) and incorporates a nonparametric estimator of the probability density function (PDF) in the form of Kernel Density Estimation (KDE). This developed real-time algorithm enables reliable assessment of vital parameters of evacuated individuals. The wristband contact with the skin is verified by measuring the brightness of backscattered light and the temperature of the wrist. Motion detection is achieved using the MPU-9250 inertial module, which analyzes acceleration across three axes. This allows the system to distinguish between states of rest and physical activity, which is crucial for accurately interpreting vital parameters during evacuation. The experimental studies, which were performed on a representative group of individuals, confirmed the correctness of the developed algorithm. The system ensures reliable monitoring of vital parameters by combining precise pulse detection, skin contact verification, and motion analysis. The classifier achieves nearly 95% accuracy and an *F*_1_-score of 0.9465, which indicates its high quality. This level of effectiveness can be considered fully satisfactory for evacuation monitoring systems.

## 1. Introduction

Numerous approaches exist in the literature for non-invasive vital signs monitoring. For instance, Ref. [1] presents an algorithm based on spectral analysis of the PPG signal using Correntropy Spectral Density, enabling simultaneous estimation of heart rate and respiratory rate, which confirms the effectiveness of methods based on frequency transformations. Conversely, Ref. [2] introduces the concept of digital biomarkers derived from non-contact sensors in home environments, emphasizing the growing importance of remote health monitoring for elderly care. Another promising area is the use of acoustic signals, as demonstrated by the EarSleep system [3], which shows that analyzing sounds produced by the body can effectively aid in recognizing sleep stages, opening new avenues for monitoring physiological functions. Lastly, the technology review in [4] underscores the significance of contactless solutions in reducing infection risk and enhancing patient comfort, especially in real-time systems. The publications mentioned focus on monitoring patients through detailed analysis of vital functions. In many cases, especially for certain solutions like evacuation monitoring systems for nursing home residents, complex analyses of pulse or respiratory rate are unnecessary. Instead, it is sufficient to fast and efficiently detect the presence or absence of essential vital signs. Such a device, designed to provide simple, effective, and energy-efficient vital sign detection, is discussed below. As part of this research, an advanced electronic system was developed to support the evacuation processes of individuals with special needs. This system comprises two primary components: hardware and software. The hardware component includes designed wristbands for monitoring the location and health status of individuals, as well as locators placed within the building, integrated with a suitably selected communication system. The system was designed based on an architecture that includes wristbands assigned to each of the evacuated individual, locators installed at key points in the building, and dedicated software.

**Figure 1:**
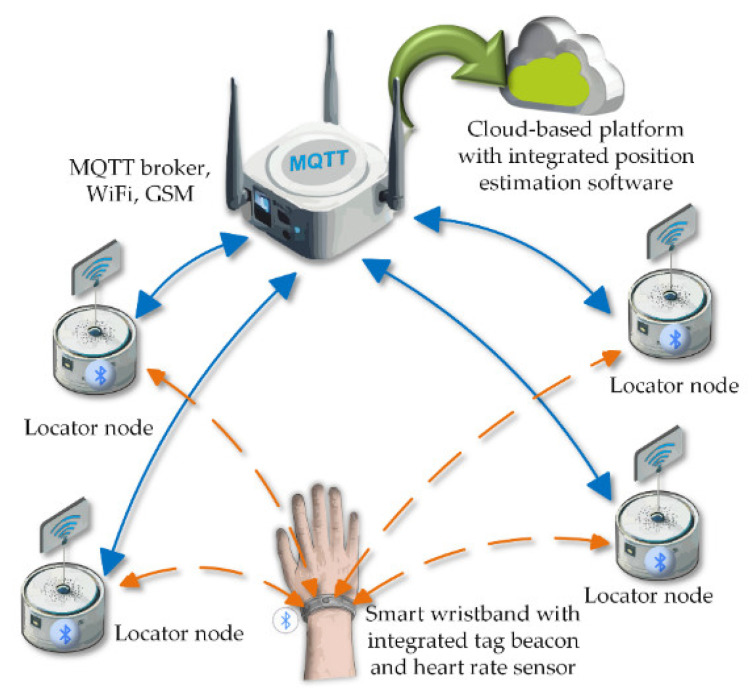
The general concept of the evacuated person location system, in which the personal wristband contains PPG and inertial measurement unit (IMU) sensors.

The core element of the system (Figure 1) is the software that integrates data transmitted by the locators, processes it in an estimation module, and presents it to the user in the form of visualizations. Additionally, the software component includes a web and mobile application, as well as a set of components responsible for interfacing with electronic devices. The project implementation utilizes technologies such as Bluetooth, WiFi, Internet of Things (IoT) solutions, and flexible, scalable software based on a microservices architecture [5]. This solution is primarily dedicated to institutions such as nursing homes, hospitals, schools, or public offices, where it can effectively support the monitoring of individuals requiring special care.

Systems like the one proposed in Figure 1 can incorporate various technical solutions to enhance monitoring, such as tracking vital functions. Modern wearable devices are becoming a critical component in real-time vital signs monitoring [6,7,8,9,10], providing essential support in crisis situations, such as evacuation from hazardous areas. Key functionalities may include the measurement of heart rate (HR), blood oxygen saturation (SpO_2_), respiratory rate (RR), heart rate variability (HRV), as well as skin temperature and blood pressure [11,12,13].

The most common technology for HR and HRV detection in wearable devices is photoplethysmography (PPG), which relies on analyzing changes in blood volume in microcirculation registered using LEDs and photodetectors. This method allows for continuous measurement of the above parameters under dynamic conditions such as movement or stress, although its accuracy may be limited in these cases [13,14,15].

For monitoring blood oxygen saturation (SpO_2_), multi-spectral PPG is used with red and infrared light to estimate the oxygen saturation of hemoglobin [16]. This non-invasive method requires careful calibration and consideration of environmental factors, which can be challenging in evacuation conditions [17].

Respiratory rate (RR) measurement is typically performed indirectly by analyzing modulation of the PPG signal or changes in accelerometer data related to chest movements [18]. Models that fuse these signals are also used to improve accuracy under high-stress or intense physical activity [13,15]. Selected devices include methods for estimating blood pressure (e.g., using Pulse Transit Time, PTT), combining PPG with an accelerometer or electrocardiogram (ECG) [19]. While this technology seems to be promising, it requires further validation studies in real-world conditions.

Skin temperature is measured using miniature thermistor sensors; however, users should note that these primarily indicate surface temperature and require correction algorithms to relate them to core body temperature.

The use of sensors in wearable devices enables dynamic monitoring of vital functions during evacuations, allowing for the detection of health deterioration (e.g., tachycardia, hypoxia) and supporting rescuer decisions. The key challenge remains measurement accuracy under extreme environmental conditions, integration with location and communication systems, and resistance to motion artifacts [20]. In the context of evacuation, heart rate sensors are among the most frequently used as they allow for physiological monitoring, stress assessment, and early detection of health risks. For evacuations where mobility, comfort, and rapid response to changing health status are crucial, PPG sensors appear to be the most practical provided they are supported by algorithms that compensate for motion-related errors. While ECG offers higher accuracy, its use in dynamic evacuation conditions may be limited due to electrode contact requirements. PTT has potential for blood pressure monitoring but is less suitable for emergency situations due to interference sensitivity and implementation complexity.

The most common PPG sensors used in wearable devices are presented in Table 1.

Additional sensors that support vital function monitoring in wearable devices are Inertial Measurement Unit (IMU) modules enabling the measurement of motion parameters and orientation in space. These units include accelerometers measuring linear acceleration, gyroscopes measuring angular velocity around their axes, and magnetometers determining magnetic field strength. Each sensor is 3-axis and oriented in a Cartesian coordinate system. The use of IMUs involves reading raw data from the sensors and processing it through estimation algorithms, such as Kalman filters, to obtain precise information about orientation and dynamics in real time. Consequently, IMU sensors allow for monitoring body movement and gait analysis [29]. The most commonly used IMUs in consumer devices are presented in Table 2.

To achieve a more comprehensive and reliable assessment of an evacuee’s condition, the project integrates data from heart rate sensors and IMU modules. The complementarity of data provided by these sensors is particularly valuable in this application. Heart rate sensors monitor essential vital functions such as pulse frequency, which directly indicates health status. Moreover, IMUs track body movement and position, enabling detection of motion, falls, or immobility. Combining these datasets enables immediate and accurate situation assessment, especially during critical evacuations. For example, the absence of both pulse and movement may indicate a life-threatening condition, a pulse with no movement could suggest rest or temporary immobilization, and movement without a detectable pulse would immediately raise concerns. This integrated approach significantly reduces false alarms compared to monitoring single parameters alone. From an engineering perspective, both sensor types are available in compact, power-efficient forms that can be easily combined into a single device without substantial size or complexity increases. As a result, integrating heart rate sensors and IMUs creates a more comprehensive, reliable, and effective tool for monitoring the vital functions of evacuees. However, realizing this task requires developing specialized algorithms, particularly for detecting pulses, tracking movement, and fusing data within the limited resources of a wearable device.

## 2. Objective and Contributions of the Paper

The primary objective of this article is to present a pulse detection algorithm that is robust against dynamic disturbances caused by wristband vibrations, commonly occurring when people are moving during evacuation or experiencing tremors due to other medical conditions. It is essential to note that this algorithm primarily focuses on detecting the presence of a pulse, rather than analyzing its characteristics. From the perspective of first responders and the subsequent evacuation process, confirming whether someone is alive and prioritizing their immediate extraction from danger is critical. This publication details an effective approach for pulse detection specifically designed to operate in real time in environments with increased mechanical interference. The proposed solution effectively eliminates disturbances caused by movement, particularly during dynamic events like building evacuations. Furthermore, the algorithm accounts for uncontrolled hand movements that may result from neurological conditions or other impairments frequently seen in elderly and disabled individuals. Additionally, the goal of the developed algorithm is to operate in real time while maintaining the lowest possible LED brightness level, which significantly reduces energy consumption, a crucial factor for wearable devices. By employing a robust signal analysis method, this pulse detection solution integrated with IMU system provides reliable monitoring of vital signs even under non-static conditions. This approach features the following:

- Simple implementation.
- Low power consumption for wearable devices.
- Compatibility with microcontrollers that have limited processing capabilities.
- Real-time performance enabling accurate pulse detection.

These characteristics make the algorithm particularly suitable for emergency applications where portability, efficiency, and reliability are paramount.

## 3. Detection of Vital Functions Using a Pulse and Blood Oxygen Sensor

One of the most popular PPG sensors is the MAX30102, an advanced integrated solution combining red (660 nm) and infrared (880 nm) LEDs, a photodetector, and optical components in a single compact module. This design makes it ideal for wearable applications where size and weight are critical concerns. The MAX30102 is characterized by low power consumption, enabling extended monitoring without frequent charging. Integrated signal filtering and noise reduction mechanisms, including motion artifact cancelation, ensure measurement accuracy even under dynamic-use conditions. As a multispectral sensor, it simultaneously tracks heart rate and SpO_2_ delivering data through a single I^2^C interface. Its widespread availability and competitive pricing make the MAX30102 a cost-effective choice, often selected by wearable device manufacturers.

The MAX30102 sensor is widely supported by available programming libraries and technical documentation, which facilitates its integration and accelerates the prototyping process. The sensor driver allows the adjustment of parameters related to both the emission of light from the diodes and the processing of signals from the photodetector. The library [37] enables modification of parameters such as LED brightness, averaging window width, selection of the active LED type, sampling frequency, pulse duration (LED emission time expressed in µs), and the measurement range of the ADC converter. The example of the parameter setup can be presented as follows:

byte ledBrightness//Options: 0 = Off to 255 = 50 mA
byte sampleAverage//Options: 1, 2, 4, 8, 16, 32
byte ledMode//Options: 1 = Red only, 2 = Red + IR, 3 = Red + IR + Green
int sampleRate//Options: 50, 100, 200, 400, 800, 1000, 1600, 3200
int pulseWidth//Options: 69, 118, 215, 411 us
int adcRange//Options: 2048, 4096, 8192, 16,384 nA

The backscattered light signal serves as the primary component containing the pulse wave from which the heart rate can be determined. The goal of this research was to develop algorithms for pulse detection that would maintain required accuracy while minimizing energy consumption. Preliminary studies identified LED brightness (ledBrightness) and pulse width (pulseWidth) as key parameters influencing both detection quality and power consumption. The measurement of the current of the IR diode confirmed the linear nature of the relationship between the value of the ledBrightness parameter and the current flowing through the LED. For example, for a ledBrightness setting of 10, the measured current value was 1.8 mA, while a ledBrightness value of 190 corresponded to an LED current of 32.21 mA. To determine optimal values for the mentioned parameters, a series of experiments was performed. The experiments involved recording signals from the MAX30102 sensor. During the measurements, the IR LED brightness was increased every 5 s in steps of 10, from 10 to 200. The RED LED served as an indicator with a brightness of 1 for a possible adjustable range of 1–255. With this setup, the diode selection parameter was set to ledMode = 2.

**Figure 2:**
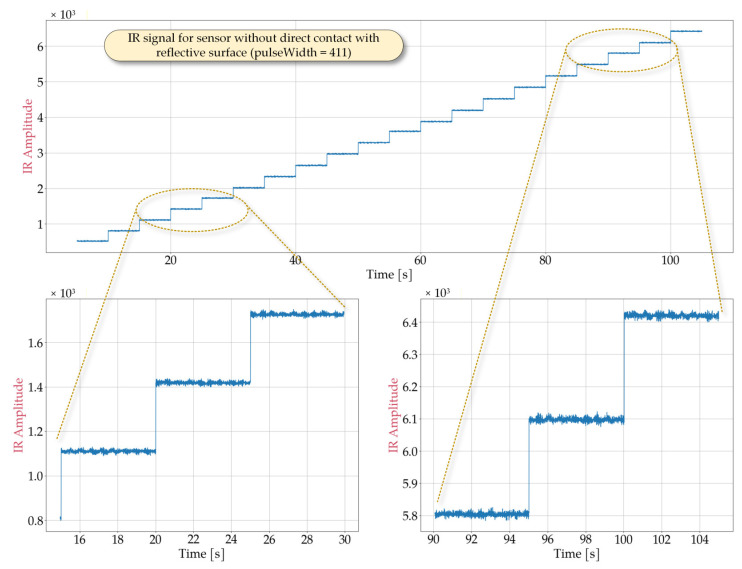
Waveforms obtained from the MAX30102 sensor lying freely, without contact with the reflective surface (oriented with the LED side facing upward) during a measurement in which the IR ledBrightness was increased every 5 s from 10 to 200 in steps of 10 and pulseWidth set to 411.

**Figure 3:**
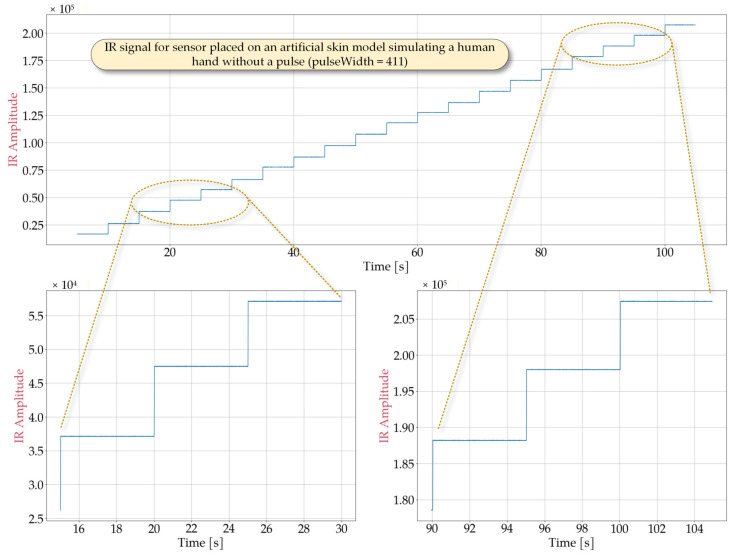
Waveforms obtained from the MAX30102 sensor placed on an artificial skin model simulating a human hand without a pulse during a measurement in which the IR ledBrightness was increased every 5 s from 10 to 200 in steps of 10 and pulseWidth set to 411.

**Figure 4:**
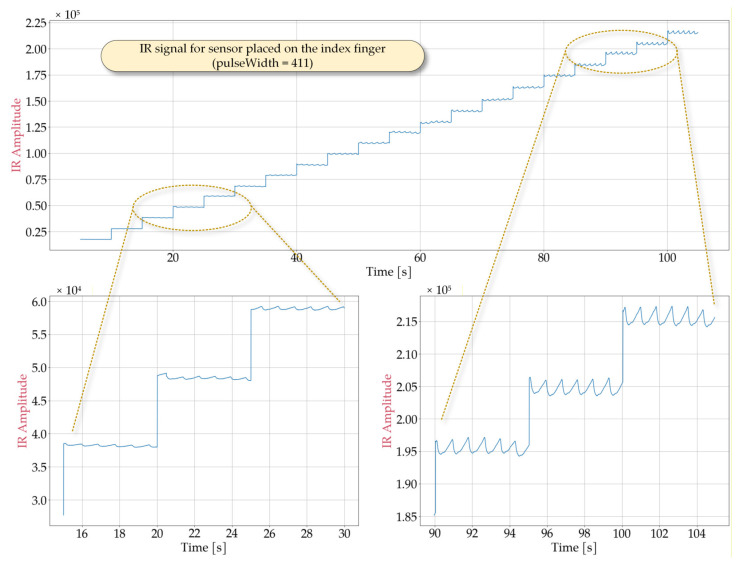
Waveforms obtained from the MAX30102 sensor placed on the index finger during a measurement in which the IR ledBrightness was increased every 5 s from 10 to 200 in steps of 10 and pulseWidth set to 411.

**Figure 5:**
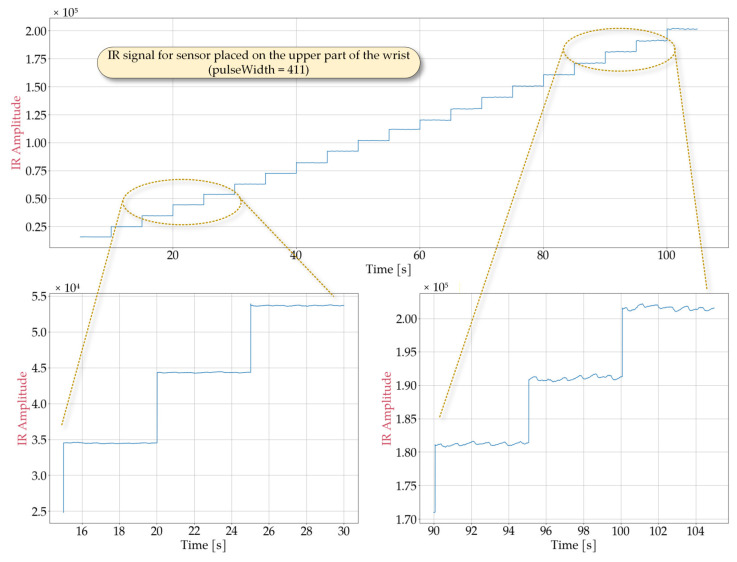
Waveforms obtained from the MAX30102 sensor placed on the upper part of the wrist during a measurement in which the IR ledBrightness was increased every 5 s from 10 to 200 in steps of 10 and pulseWidth set to 411.

In order to determine optimal sensor performance under various conditions, laboratory tests simulating potential cases of use were conducted. Representative signal obtained from the MAX30102 sensor are shown in Figure 2, Figure 3, Figure 4 and Figure 5 for the following cases: sensor positioned freely without contact with a reflective surface, sensor is inverted with LEDs facing upwards (Figure 2), measurement on an artificial skin model mimicking a human hand without a pulse (Figure 3), measurement on a fingertip (Figure 4), measurement on a dorsal wrist area (Figure 5). All measurements were maintained at the following settings: sampleAverage = 2, pulseWidth = 411 µs, and sampleRate = 1000 Hz.

The experimental conditions for Figure 2, Figure 3, Figure 4 and Figure 5 were selected to represent diverse usage scenarios of the wearable device, enabling the development of robust detection algorithms. For instance, Figure 2 depicts a scenario where the device is not worn (e.g., lying on a table). As it can be observed, this configuration produces a signal with limited variation, with a maximum value not exceeding $7\times 10^{3}$. The signal exhibits noise-like behavior with low variability (ranging from 60 to 80) that is largely independent of LED intensity. However, changes in the average signal level are noted, which correlate with changes in LED brightness, suggesting minimal but detectable optical crosstalk between the source and detector. Figure 3 illustrates measurements taken on an artificial skin model simulating a human hand without a pulse, representing cardiac arrest or sudden cessation of heartbeat. This configuration also yields a noise-dominated signal with a variability of approximately 300, largely independent of the LED intensity. The average signal value shows greater variation compared to Figure 2, reaching a maximum value of roughly $2.1\times 10^{5}$. Notably, while both scenarios produce signals with similar statistical characteristics (noise-like behavior), they differ significantly in their mean levels, allowing for reliable differentiation between device malfunction or misplacement and genuine pulse absence.

The scenario presented in Figure 4, which involves pulse detection on the index finger, does not correspond to the typical wristband placement; however, it allows obtaining a clear and stable heart rate signal. As shown in the figure, the average signal value depends on the LED brightness settings, reaching approximately $2.1\times 10^{5}$ at maximum brightness. The signal takes the form of a sequence of pulses with a period corresponding to the heart rate and an amplitude dependent on the LED brightness level. The peak-to-peak value (*S_pp_*) averages between *S_pp_* = 220 for a ledBrightness setting of 20 and *S_pp_* = 2770 for a ledBrightness setting of 200, with the *S_pp_* value increasing only marginally above a ledBrightness of 130. This effect can serve as a guideline for selecting the ledBrightness parameter. This parameter directly affects energy consumption, and efforts should be made to minimize it while considering the quality of pulse detection. It is worth noting that, in this case, good signal quality enables pulse detection and heart rate determination using simple algorithms, such as those identifying local extrema. As observed in Figure 4, even at low LED brightness levels, the signal enables obtaining satisfying results using these algorithms. However, these methods are not effective for the typical wristband placement, such as the wrist. As shown in Figure 5, in such cases, the recorded pulses exhibit irregular shapes, varying peak-to-peak values, and multiple local maxima. Furthermore, at low ledBrightness values, pulse detection becomes infeasible. Consequently, there is a clear need to develop a dedicated pulse detection algorithm that operates with satisfactory detection quality at the lowest possible LED brightness.

**Figure 6:**
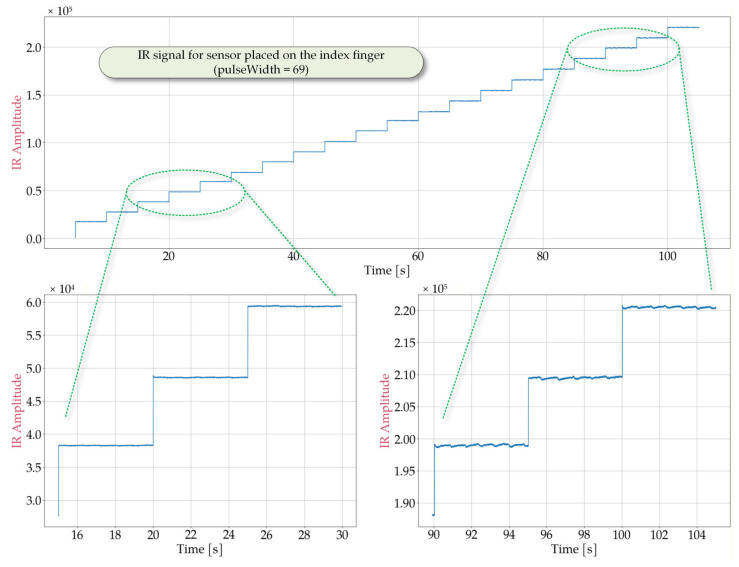
Waveforms obtained from the MAX30102 sensor placed on the index finger, with the pulseWidth set to 69 and the ledBrightness increased from 10 to 200 in steps of 10 every 5 s.

Another parameter that affects the quality of the signal from the IR detector is pulseWidth. This parameter controls the pulse width of the LED, directly influencing the clarity and peak-to-peak value of the recorded signals. This issue can be analyzed comparing Figure 4 and Figure 6. These figures present the waveforms obtained from the MAX30102 sensor during pulse detection on the index finger, with the pulseWidth parameter set to 69 (Figure 6) and 411 (Figure 4), respectively. In both cases, the sampleAverage parameter was set to 2, and the sampleRate parameter was set to 1000.

As shown in Figure 6, for pulseWidth = 69, the signal level is very low, and even at the highest LED brightness level, the signal is comparable to the noise level. This setting does not allow for reliable pulse detection when the wristband is worn. According to Figure 4, for pulses with a pulseWidth = 411, the signal consists of a series of distinct pulses with a peak-to-peak value of approximately *S_pp_* = 3000 at a high LED brightness level (ledBrightness = 200). Furthermore, even at low ledBrightness values, pulses can still be distinguished in the signal. Therefore, pulseWidth = 411 was selected.

Another parameter crucial from the perspective of the processing procedure is the sampleRate, which defines the sampling frequency. This parameter should be set to ensure both adequate temporal resolution and the bandwidth of the processed signal. Assuming a very high resolution, i.e., 250 samples per pulse at a high heart rate, and considering the pulseWidth = 411 setting, the sampleRate of 1000 was selected. This value provides sufficient bandwidth for the analysis required by the proposed detection algorithm.

Moreover, the analog-to-digital converter range, defined by the adcRange parameter, was determined experimentally for different surfaces and LED brightness levels (ledBrightness). The chosen value for this parameter is adcRange = 16,384, which protects the converter from saturation.

The parameter sampleAverage defines the number of samples to be averaged. At the given sampling frequency, setting sampleAverage = 2 allows for moderate noise reduction without causing excessive blurring of the recorded signal.

Based on the analyses presented above, the following configuration parameters for the MAX30102 sensor were established:

byte ledBrightness 170
byte sampleAverage 2
int sampleRate 1000
int pulseWidth 411
int adcRange 16,384

**Figure 7:**
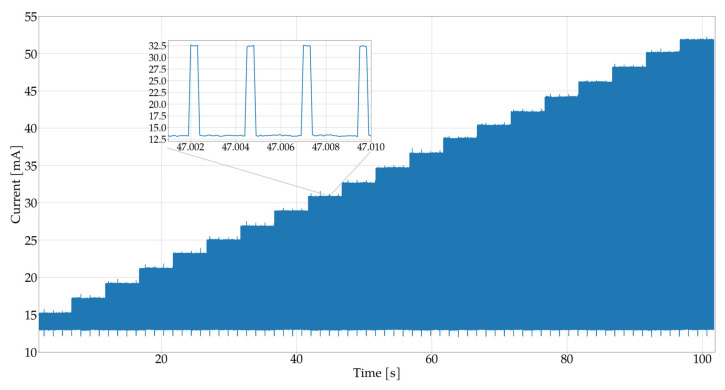
Illustration of the relationship between current consumption and the brightness of the RED LED in the MAX30102 sensor.

**Figure 8:**
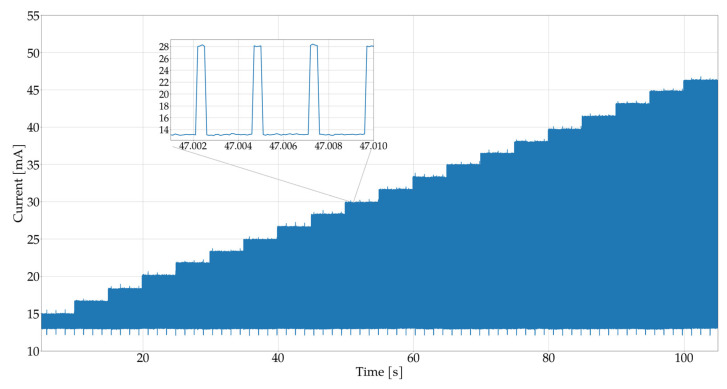
Graph of the relationship between current consumption and the brightness level of the RED IR LED in the MAX30102 sensor.

Furthermore, in order to illustrate the energy efficiency of the measurement system, an experiment was conducted for the previously selected parameters, in which the current consumed by the entire system, i.e., the microcontroller together with the MAX30102 sensor, was measured. Figure 7 and Figure 8 show the current consumed by the measurement system. During the tests, the brightness of the IR and RED LEDs was increased, as described in previous experiments, and the current consumption was measured. The results show that the relationship between LED brightness and current consumption is practically linear.

This indicates that the brighter the LED shines, the more current is consumed by the system. In the context of the proposed algorithm, the brightness of the diode can be adjusted to reduce energy consumption. The proposed pulse detection algorithm is designed to minimize current consumption. In practice, if high brightness is not required for effective detection, it can be reduced, resulting in lower energy consumption and extended battery life.

In tests conducted on two types of diodes—red and infrared—it turned out that the infrared diode has lower current consumption. Moreover, the infrared diode provides a stronger received signal, which results in more effective detection compared to the red diode. In summary, the choice of an infrared diode is beneficial both in terms of energy efficiency and better signal quality used in the device.

## 4. Vital Signs Detection Algorithm

The assumptions of the implemented system impose on the signal processing algorithm the detection of the following states, enabling the identification of vital signs crucial from the perspective of the evacuation process:

- Pulse detected
  o The person is moving—the system monitors the evacuation process.
  o The person is stationary, indicating the need for assistance during evacuation.
- No pulse detected
  o The wristband is not worn—no possibility to monitor the evacuation process.
  o Vital functions have ceased.

The implementation of the intended functions requires pulse and motion detection, as well as temperature measurement. It should be noted that in this application, there is no need to determine the exact HR, only pulse detection. Detection of pulse presence was realized using a developed signal-processing algorithm applied to the photodetector signal from the MAX30102 sensor. Motion detection was achieved by analyzing data from the IMU MPU-9250 accelerometer. The classification of pulse and motion states is performed separately and is not integrated through direct signal fusion. Due to the system assumptions, which aimed to recognize one of four possible states, the actual data integration from both sensors is performed at a higher level and consists of logically combining the classification results.

Basic methods for estimating HR based on measurements obtained from the MAX30102 sensor have been described in numerous publications [19,20], and practical implementations of selected algorithms for embedded systems can be found, for example, in [33]. However, the requirements of the developed system impose the need to provide only pulse detection, while maintaining a short response time in real time and robustness against disturbances caused by wristband vibrations on the monitored person’s wrist and minimizing power consumption.

The algorithm had to be adapted for practical use in single-chip microcontrollers with limited hardware resources and computational capabilities. Consequently, a dedicated algorithm was developed that meets the above assumptions and enables reliable operation without requiring high computational power.

In the implemented system for vital signs detection, the pulse detection algorithm is executed in several stages, presented in the form of pseudocode shown in Listing 1.

The processing begins with acquiring *N_IR_* samples of the input signal recorded by the optical sensor. The value of *N_IR_* depends on the lowest pulse rate that the system can detect. For example, for a minimum heart rate of 30 beats per minute, the duration of two full pulse cycles is approximately 4 s (*τ* = 4 s). Therefore, the number of *N_IR_* samples required for further analysis is calculated as the product of the time *τ* and the sampling frequency *fₛ* (i.e., *N_IR_* = *τ* · *fₛ*). Subsequently, the average value *S_AVG_* is computed from the acquired samples according to (1):

(1) $$S_{AVG}=\frac{1}{N_{IR}}\sum_{n=1}^{N_{IR}}S_{IR}\left(n\right)$$

where *S_IR_*(*n*) denotes the *n*-th sample of the signal from the MAX30102 sensor.

The calculated value of *S_AVG_* enables the determination of the signal level indicating the type of surface that reflects the light emitted by the IR diode. In the next step, the DC component is removed from the *S_IR_* signal according to (2):

(2) $$\;\;\;\;\;\;\;\;\;\;\;\;\;\;\;\;\;\;\;\;\;\;\;\;\;\;\;\;\;\;S_{P}\left(n\right)=S_{IR}\left(n\right)-\;S_{AVG}\;\;\;\;\;\;\;\;\;\;\;\;\;\;\;\;\;\;\;\;\;\;\;n=1,\ldots ,\;N_{IR}\;,$$

where *S_P_*(*n*) is the signal with the DC component reduced and serves as the basis for further processing.

An example of the *S_P_* waveform obtained from the MAX30102 sensor after DC component removal is presented in Figure 8. It is important to note that this signal is measured at a lower brightness level of the IR diode. In this scenario, trying to determine heart rate frequency directly from the time-domain waveform is challenging and may lead to errors, particularly when dynamic disturbances are present.

**Listing 1.** Pseudocode of the pulse detection algorithm.**INPUT** min_BPM ← 30      *//minimal heart rate* f_s ←1000         *//Sampling frequency [Hz]* T_h ← 10000       *//value of the pulse detection threshold* **CONSTANTS** tau ← (60/min_BPM) * 2 * //Duration of two heartbeats [s]* N_IR ← tau * f_s       *//Number of samples to acquire* f_min ← 0.5         *//Minimal detected pulse frequency [Hz] (30 BPM)* f_max ← 4.0        *//Maximal detected pulse frequency [Hz] (240 BPM)* **BEGIN** ***//1. Acquire N_IR samples from the sensor***     signal ← acquire_signal (n) ***//2. Calculate mean of the signal***     S_AVG ← mean (signal) ***//3. Filter signal: remove DC component (subtract mean)***     filtered_signal ← signal – S_AVG ***//4. Compute Fourier Transform of filtered signal***     spectrum ← FFT (filtered_signal) ***//5. Isolate frequency range corresponding to typical heart rate***     pulse_band ← get_frequency_range (spectrum, f_s, f_min, f_max) ***//6. Find frequency with maximum amplitude in the pulse band***     S_MAX ← find_max_amplitude_frequency (pulse_band) ***//7. Decision: check if detected frequency is in valid heart rate range***     IF S_MAX ≥ T_h THEN      pulse_detected ← TRUE     ELSE      pulse_detected ← FALSE     ENDIF ***//8. Return result***     RETURN pulse_detected, S_MAX **END**


The *S_P_* signal is then subjected to spectral analysis using the Fast Fourier Transform (FFT), which is a computationally efficient implementation of the discrete Fourier Transform (3):

(3) $$S_{F}\left(m\right)=\sum_{n=0}^{N_{IR}-1}S_{P}\left(n\right)e^{-j\frac{2\;\pi }{N_{IR}}\;m\;n}$$

where *S_F_*(*m*) is the *m*-th frequency component of the *S_P_* signal spectrum.

**Figure 9:**
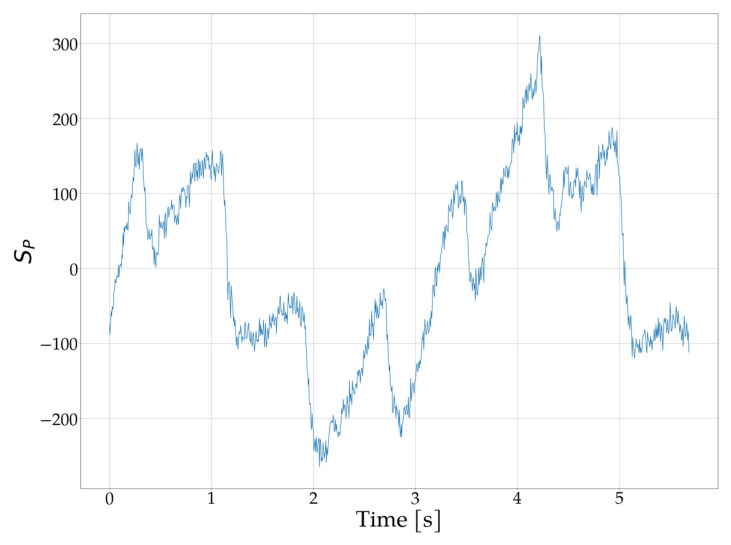
Waveform *S_P_* obtained from the MAX30102 sensor placed on the upper part of the wrist after reduction in the DC component.

**Figure 10:**
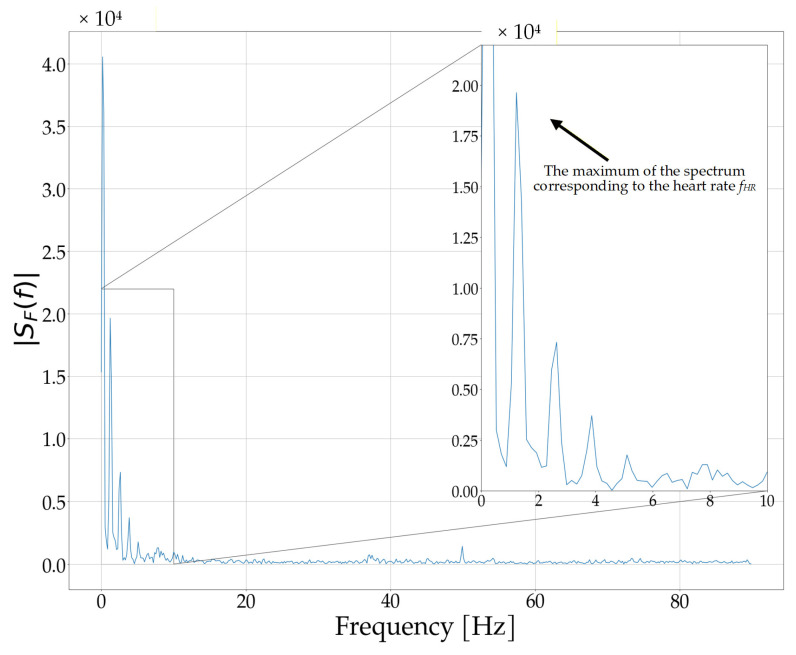
Amplitude spectrum of the *S_P_* signal, used for coarse heart rate frequency *f_HR_* estimation.

Using this approach, the pulse detection can be performed by analyzing the spectral amplitude peaks, rather than through time-domain analysis of the signal subjected to complex bandpass filtering. The amplitude spectrum of the *S_P_* signal shown in Figure 9 is presented in Figure 10. As shown in the figure, disregarding the spectrum of the residual DC component, the peak of the spectrum approximately corresponds to the pulse frequency *f_HR_*.

From the obtained spectrum, the position of the maximum amplitude within the frequency range $\langle f_{min},f_{max}\rangle$ corresponding to the expected heart rate frequencies is determined, allowing for a coarse estimation of the heart rate frequency $f_{HR}$ (4):

(4) $$f_{HR}=\underset{f_{min}\leq f\leq f_{max}}{arg\;max}\left|S_{F}\left(f\right)\right|,\;\;\;\;\;\;\;\;\;\;\;\;f=m\;\frac{f_{s}}{N_{IR}},$$

The amplitude value of the spectrum $S_{max}=\left|S_{F}\left(f_{HR}\right)\right|$ at the determined frequency $f_{HR}$ forms the basis for the final stage of the algorithm, which is the decision whether the signal contains signs of cardiac activity (pulse presence) (hypothesis $\;H_{1}$) or not (hypothesis $H_{0}$). In the developed algorithm, pulse presence detection is based on statistical detection theory. The decision on pulse presence is made based on the probability density function $p_{S_{max}}\left(S_{max}\right)$ of the variable $S_{max}$ and a decision threshold $T_{h}$, which is determined according to the assumed miss detection probability $P_{MD}$ [34]. The detection threshold was established based on (5).

(5) $$P_{MD}=P_{r}\left(S_{max}\;\leq \;T_{h}|H_{1}\right)=\int_{S_{max}\;\leq \;T_{h}\;}p_{S_{max}}\left(S_{max}\right)\;{dS}_{max}$$

The probability density function $p_{S_{max}}\left(S_{max}\right)$ was estimated using kernel density estimation (KDE) [35,36]:

(6) $$\hat{f_{h}}\left(x\right)=\frac{1}{\sqrt{2\pi }\;nh\sigma }\sum_{i=1}^{n}\exp\left(-\frac{{\left(x-x_{i}\right)}^{2}}{2h^{2}\sigma^{2}}\right),$$

where $\hat{f_{h}}\left(x\right)$ is the estimated probability density function, *n* is the number of samples, *h* is the kernel bandwidth, $x_{i}$  *i*-th data sample, and *σ* is the standard deviation of the variable *x*. In the case of the proposed pulse detection algorithm, the Scott’s Rule was used to determine the *h* parameter [36].

**Figure 11:**
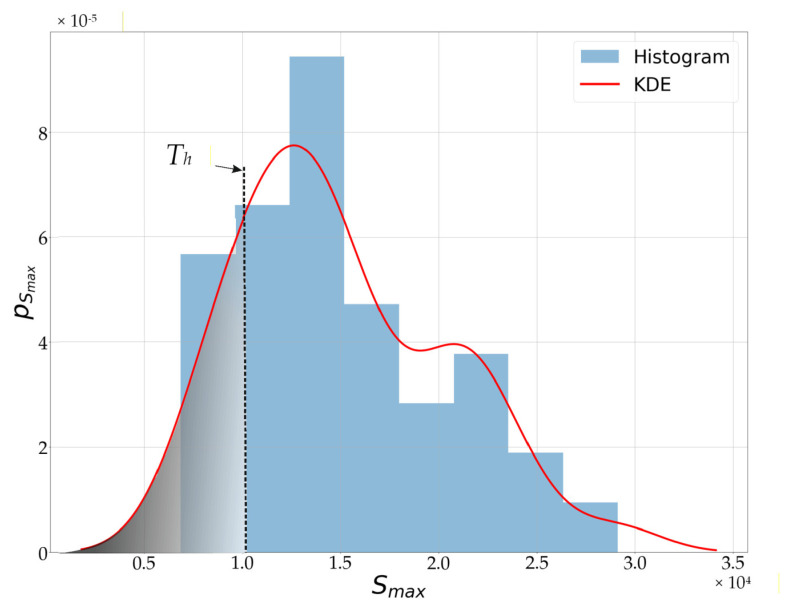
Estimation of the probability density function $p_{S_{max}}\left(S_{max}\right)$ and an illustration of the threshold *T_h_* selection, for the signal obtained from the MAX30102 sensor, located on the top of the wrist.

Figure 11 presents a histogram of the $S_{max}$ variable values along with the estimated probability density function $p_{S_{max}}\left(S_{max}\right)$ obtained using the nonparametric KDE estimator. The figure also illustrates the selection of the threshold $T_{h}$, which, for the studied case, was set corresponding to a miss detection probability $P_{MD}=0.2$.

Pulse presence detection involves determining the following hypotheses: $H_{1}$—pulse is present, and $H_{0}$—no pulse is present. The decision is made by comparing the value of the variable $S_{max}$ with the decision threshold $T_{h}$, in accordance with (7):

(7) $$\mathrm{Pulse}\;\mathrm{detection}=\left\{\begin{array}{cc} H_{1}:pulse\;\mathrm{detected}\; & \mathrm{if}\;S_{\max}\geq T_{h}\\ H_{0}:\mathrm{pulse}\;\mathrm{not}\;\mathrm{detected}\; & \mathrm{if}\;S_{\max}<T_{h} \end{array}\right.$$

The efficiency of the proposed pulse detection algorithm was verified using data obtained from Pulse Transit Time PPG Dataset [37,38]. This database contains recordings of signals from the MAX30101 PPG sensor. The dataset comprises measurements collected from 22 individuals across three states of physical activity: resting, walking, and running. The PPG sensor signals were recorded at a sampling rate of 500 Hz for about 8 min.

According to the procedure proposed in the article, the infrared wavelength PPG measurements were divided into 4 s time segments. Half of the resulting data was used to estimate the probability density function and determine decision thresholds, as described in Equations (1)–(6). The remaining data was used as test data to evaluate the effectiveness of the algorithm presented in Listing 1. Both datasets were selected from randomly chosen segments.

**Figure 12:**
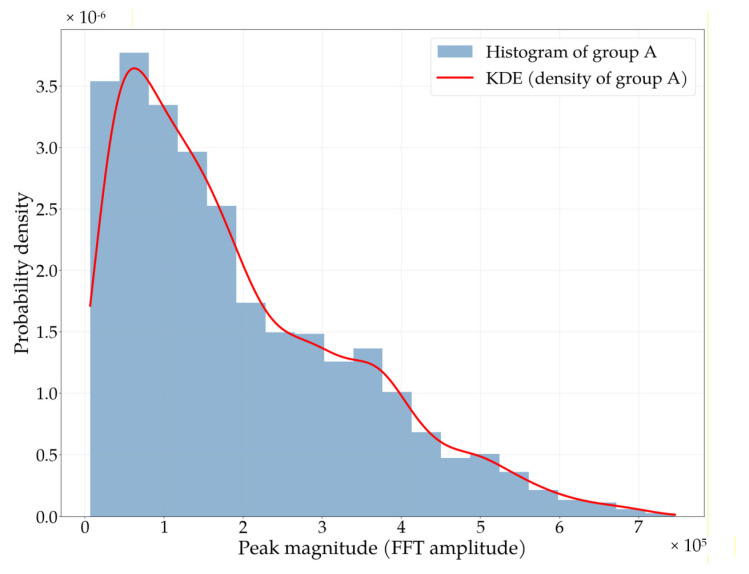
Estimate of the probability density function obtained for data from the database [38].

The Python 3.11 software [39] was used to implement and test the algorithm. In the scripts, signal processing is carried out in the previously described stages. The first step is to remove the signal’s DC component using a high-pass filter, as described by (1) and (2). Next, frequency analysis (3) is performed using the FFT method for each 4 s fragment. In the FFT spectrum, the first maximum corresponding to the pulse frequency is located using (4). Based on the first dataset, a probability density function is estimated using the KDE method (6). Then, a decision threshold is determined according to (5) for the assumed $P_{MD}$. Estimates of the probability density function for data obtained from the database [38] are shown in Figure 12.

Using the test data, the effectiveness of the algorithm shown in Listing 1 was evaluated. During testing, several $P_{MD}$ values were assumed, and decision thresholds $T_{h}$ were determined based on the distribution shown in Figure 10. Then, for those thresholds, the pulse detection probability was determined using the test data. The results are presented in Table 3. As shown, there was a strong correlation between assumed $P_{MD}$ and $P_{MD}$ obtained during testing on the test data. In the implemented evacuation monitoring system, a value of $P_{MD}=0.2$ was adopted, as required by the project assumptions. However, during the study, we also examined other $P_{MD}$ levels ranging from 0.005 to 0.2 (assumptions made by the system designer), which may be relevant for different types of projects. The results presented in Table 3 confirm the high efficiency of the proposed pulse detection algorithm across diverse physical activities performed by participants and demonstrate its potential applicability in other system designs.

**Figure 13:**
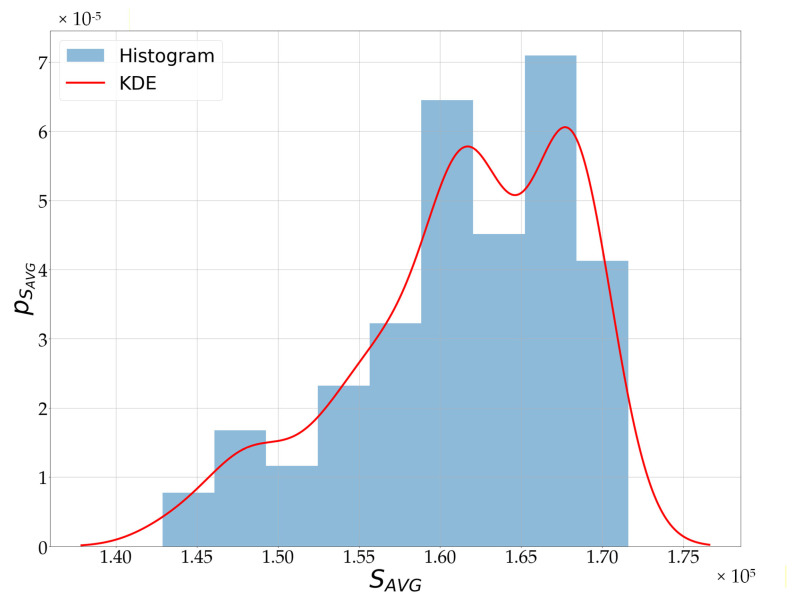
Estimation of the probability density function $p_{S_{AVG}}\left(S_{AVG}\right)$ for the signal obtained from the MAX30102 sensor, located on the top of the wrist.

It is worth noting that the next step after the pulse detection algorithm, though not essential, is to determine whether the wristband is being worn by the user. For this purpose, the signal recorded by the photodetector is analyzed. This signal depends on the intensity of light backscattered from the surface beneath the wristband. To analyze the brightness of the backscattered light, the probability density function estimate $p_{S_{AVG}}\left(S_{AVG}\right)$ of the variable $S_{AVG}$ (1) is used. The estimate $p_{S_{AVG}}\left(S_{AVG}\right)$ was obtained using a non-parametric KDE estimator. The resulting estimate and the histogram of the $S_{AVG}$ variable values are shown in Figure 13.

The estimate of the function $p_{S_{AVG}}\left(S_{AVG}\right)$ enables the determination of decision thresholds in a manner analogous to (5). The decision on whether the wristband is being worn by the user is made based on whether the current value of $S_{AVG}$ falls within a defined range between two thresholds: a lower and an upper one.

If $S_{AVG}$ is below the lower threshold, it indicates that the wristband is not in contact with the skin and that the light is being reflected, for example, from a dark background. Conversely, if it exceeds the upper threshold, the light is reflected from a bright surface, which also indicates a lack of contact with the body. Only when the backscattered light value falls between these thresholds can it be assumed that the wristband is being worn.

It is important to note that the backscattered light value also depends on individual user characteristics, such as skin tone, which is why the system must account for a certain personal margin. Based on the average brightness of the backscattered light, the presence of skin contact can be preliminarily assessed.

If the system does not detect a pulse, it does not necessarily mean that the wristband has been removed. It may also indicate that the pulse is not being detected. It may still be worn, but it may be too loose, or it may be resting on another surface. To improve the reliability of detecting such cases, the system also uses a temperature sensor (NTC), which additionally measures body temperature at the point of contact with the wristband. Therefore, to conclusively determine whether the wristband is being worn, the system employs two simultaneous conditions: an appropriate reflection value and a corresponding body temperature.

In the implementation of the evacuation monitoring system, the threshold value $T_{ht}=27\;{}^{\circ}C$ was adopted. This value was derived as the midpoint between the average temperature measurements for the wristband when worn and when lying freely. The final decision regarding whether the wristband is being worn is based on the simultaneous fulfillment of two conditions: the measured temperature must exceed the $T_{ht}$ threshold value, and the current value of $S_{AVG}$ (1) falls within a range between two thresholds.

In the process of vital signs detection, it is necessary to detect the movement of the evacuated person. For this purpose, the MPU-9250 sensor was used. This sensor, as an integrated IMU module, is equipped with a three-axis gyroscope, accelerometer, and magnetometer. The motion detection algorithm records acceleration data from the accelerometer along three axes: *X*, *Y*, and *Z*. The data is recorded in time segments equal to the time intervals *τ* used for analyzing data from the photodetector. Based on the values of the *X*, *Y*, and *Z* acceleration components, a numerical indicator *S* (8) is calculated, which serves as a measure of motion:

(8) $$S=\frac{1}{N_{acc}}\sum_{i=0}^{N_{acc}-1}\left(\left|\bar{x}-x_{acc}\left(i\right)\right|+\left|\bar{y}-y_{acc}\left(i\right)\right|+\left|\bar{z}-z_{acc}\left(i\right)\right|\right),$$

where $x_{acc}\left(i\right),\;y_{acc}\left(i\right),\;z_{acc}\left(i\right)\;$ are values from a buffer of length $N_{acc}$ containing accelerometer data, whereas $\bar{x},\;\bar{y},\;\bar{z}$ are average values calculated as follows:

(9) $$\bar{x}=\frac{1}{N_{acc}}\sum_{i=0}^{N_{acc}-1}x_{acc}\left(i\right),\;\;\;\;\;\;\;\;\;\bar{y}=\frac{1}{N_{acc}}\sum_{i=0}^{N_{acc}-1}y_{acc}\left(i\right),\;\;\;\;\;\;\;\;\;\bar{z}=\frac{1}{N_{acc}}\sum_{i=0}^{N_{acc}-1}z_{acc}\left(i\right).$$

For the proposed system, a buffer size of *N_ACC_* = 8 and an observation time of T = 4 s were established.

The value of the coefficient *S* indicates the level of physical activity detected by the sensor. Motion detection is performed by comparing the indicator *S* with an empirically determined threshold value. Exceeding the threshold results in a decision that motion has been detected.

It should be emphasized that the pulse detection and motion detection algorithms presented in the article are universal in nature and are not related only to these sensor models. The algorithms were designed to be applicable also with other sensors with similar technical characteristics. Thus, the presentation of the MAX30102 and MPU-9250 acts as an example to illustrate the operation of the method, rather than a limitation on hardware selection.

## 5. Experimental Studies on Vital Signs Detection

The effectiveness of the developed algorithms, described in Section 3 and Section 4, was verified through experimental studies conducted within a custom-built system for locating evacuated individuals. The studies related to vital signs detection constituted a separate research thread within this installation, which was primarily focused on tracking the positions of evacuated persons [40,41,42].

**Figure 14:**
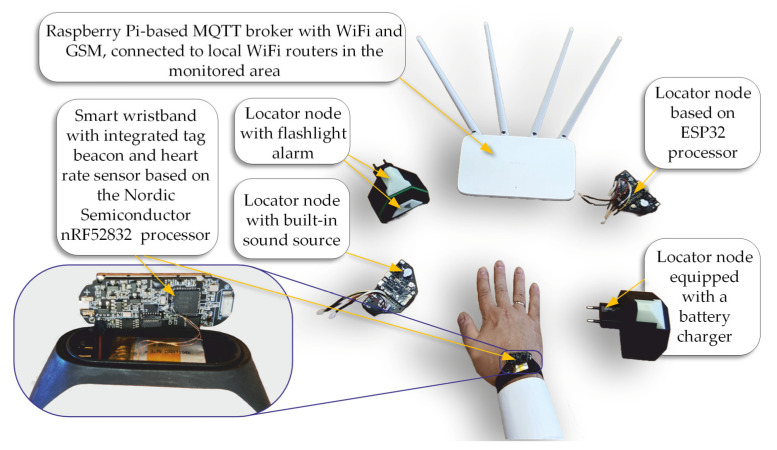
Diagram showing the implementation of the system concept introduced in Figure 1, with images of real hardware components used in the final version of the evacuation supervision system.

As part of this research, an evacuation monitoring system was developed (Figure 14), whose architecture is based on Internet of Things (IoT) technology. The system consists of a set of locators using ESP32 microcontrollers, a communication gateway, and an MQTT protocol broker installed on a Raspberry Pi single-board computer. A key component of the system is a wristband worn by the user, which functions as a mobile measurement node. The wristband is equipped with an nRF52832 microcontroller, based on an Arm Cortex-M4 core with a floating-point coprocessor, enabling efficient real-time data processing. The device supports Bluetooth 5.0 wireless communication, offering extended range and energy efficiency, which is crucial for mobile applications. To monitor the user’s motion parameters, the wristband includes an integrated MPU9250 module, which combines three types of sensors: a three-axis accelerometer, a three-axis gyroscope, and a three-axis magnetometer. The accelerometer allows for the measurement of linear acceleration in the ranges of ±2, ±4, ±8, or ±16 g, with a maximum sampling rate of 4 kHz. The most crucial component from the perspective of monitoring the user’s physiological state is the MAX30102 pulse oximeter module. This device enables non-invasive HR measurement; however, in the described case, the system does not record the absolute heart rate (expressed in beats per minute) but only detects the presence of a pulse signal. This means that the system identifies only whether a pulse signal is present or absent, without performing quantitative analysis.

One of the key functions of the designed system [43] presented in Figure 14 is the ability to estimate the location of moving Bluetooth signal sources. This task is performed by a special estimator module, which performs several important functions [44]. First, it allows the user to enter actual room plans and the exact location of Bluetooth locators. It then allows the user to add identifiers for the signal sources to be located. The estimator receives RSSI signal strength measurements from the locators using the MQTT communication protocol. Based on the data on the location of the locators and the signal strength values measured by them, the module estimates the current position of moving people. In addition, the estimator uses the MQTT protocol to transmit data on the location of evacuated persons, which allows for real-time monitoring of their position in the surveillance application.

The estimates of the locations of the wristbands and their statuses are determined based on data received from individual locators. The estimator receives data sent from locators in the ‘<main>/tracking’ topic. The message is in text format and contains 6 fields separated by semicolons:

Time (Unix epoch)
Locator MAC address
Wristband MAC address
lifesign (59000X00), where:
X = 0 → dead, not moving
X = 1 → dead, moving
X = 2 → alive, not moving
X = 3 → alive, moving
Signal level in dBi
Battery charge level in volts
An example message looks like this:
1670444444;48:53:80:56:6A:80;29:FC:15:9E:DD:6F;59000300;−57;3.2

A full description of the system and the results of tests confirming the effective operation of the complete evacuation system can be found in the papers [43,44].

An essential aspect of this research was the evaluation of the algorithm’s effectiveness. The conducted experiments focused on analyzing the accuracy of vital signs detection, with particular emphasis on pulse and human motion detection algorithms. In practical implementation, a pulse sensor worn on the user’s wrist was used, while motion detection relied on data from a three-axis accelerometer. The sensor data was processed using a dedicated pulse and motion detection algorithms. The integration of data from the pulse and motion detection modules aimed to enable classification of the physiological state and activity of the evacuated person, which constituted a key functional element of the system. It should be emphasized that despite significant disturbances in pulse signal readings caused by the user’s movement, the device was expected to meet the quality criterion defined in the project, namely, detecting vital signs with a minimum accuracy of 80%.

As part of the study, tests were conducted to evaluate the effectiveness of the vital signs detection algorithms on a representative group of individuals. Due to the planned implementation of the system, in the research presented here, the test group was selected as representative of people in nursing homes residing in north-eastern part of Europe. This was a group of 21 people of different genders, aged 50–60 years, with a fair complexion of varying shades. Approximately 30 tests were conducted for each person for each scenario. The research focused on identifying four scenarios:

- Pulse detected and motion detected, indicating that the person wearing the wristband is moving;
- Motion detected but no pulse detected—occurs when the wristband is being moved but not worn on the wrist;
- Pulse detected but no motion detected—when the person wearing the wristband remains still;
- No pulse and no motion detected—when the wristband is not worn and not being moved.

The results of the study are presented in Table 4, showing the classification accuracy for the four analyzed cases. In the developed system, data was transmitted using the MQTT protocol. The person’s state was encoded in the communication protocol using a variable that could take values from 0 to 3, as follows:

- 0: no vital signs detected, no motion detected;
- 1: no vital signs detected, motion detected;
- 2: vital signs detected, no motion detected;
- 3: vital signs detected, motion detected.

The analysis of data concerning the condition of the test group indicates varied results depending on the tested scenarios. In the resting state with the wristband worn on the wrist, pulse readings without motion dominate (81.09%), confirming the correct operation of the device in this scenario. However, a small percentage (5.02%) shows both pulse and motion, which may be the result of disturbances or slight vibrations of the wristband on the hand. When the wristband is not worn and remains stationary, all readings (100%) indicate no pulse or motion detection, which is consistent with the actual state. In the motion state with the wristband worn on the wrist, the majority of readings (92.66%) correctly detect both pulse and motion, demonstrating the effectiveness of the device during activity. Conversely, in the motion state with the wristband not worn on the wrist, all readings (100%) show motion detected and no pulse detected, which is the expected outcome.

The confusion matrix, summarizing the study, is presented in Table 5. The data presented in the table are based on four implemented scenarios.

The confusion matrix illustrates the effectiveness of the classifier responsible for detecting vital signs, depending on whether a wristband was placed on the wrist. The classifier was designed with an acceptable missed detection rate of 20%, meaning situations in which the system fails to detect vital signs even though they are present. The results show that when the wristband was worn, the classifier performed effectively. It correctly detected vital signs in 89.84% of cases. This means that it failed to detect vital signs in 10.16% of cases, which is better than the assumed threshold level. This indicates satisfactory system performance. The detection error does not exceed the established limits, confirming the proper functioning of the detection and classification algorithm. Based on the relationships between correct (*TPs*—True Positives, *TNs*—True Negatives) and incorrect (*FPs*—False Positives, *FNs*—False Negatives) classifications, the following quality metrics of the classifier were also determined:

Sensitivity (*TPR*—true positive rate)—the classifier’s ability to detect cases where a pulse is present:

(10) $$TPR=\frac{TP}{TP+FN}\cdot 100\%\approx 89.84\%$$

Specificity (*TNR*—true negative rate)—the classifier’s ability to correctly detect the absence of a pulse:

(11) $$TNR=\frac{TN}{TN+FP}\cdot 100\%=100\%$$

Precision (*PPV*—positive predictive value)—a measure of how many instances labeled as “pulse present” truly reflect the presence of a pulse:

(12) $$PPV=\frac{TP}{TP+FP}\cdot 100\%=100\%$$

Accuracy (*ACC*)—the proportion of correct classifications:

(13) $$ACC=\frac{TP+TN}{TP+TN+FP+FN}\cdot 100\%\approx 94.94\%$$

*F*_1_-score—harmonic mean of precision and sensitivity:

(14) $$F_{1}=\frac{2TP}{2TP+FP+FN}\approx 0.9465$$

The classifier reliably identifies the presence of the wristband. It has high precision and specificity (100%), meaning it does not show the presence of the wristband when it is not actually worn. The sensitivity is 90%, indicating that in 10% of cases, the system fails to detect a pulse despite the wristband being worn, which is still better than the assumed miss detection rate. The classifier achieves nearly 95% accuracy and an *F*_1_-score of 0.9465, which indicates its high quality. This level of effectiveness can be considered fully satisfactory for evacuation monitoring systems.

It should be noted that during testing, proper placement of the wristband on the wrist was found to be crucial for the reliability of measurements in both resting and motion states.

In summary, the results of the conducted study confirm the correct operation of both the wristband and the developed software, as well as the effectiveness of the implemented detection algorithms.

## 6. Discussion

This article aims to develop an efficient algorithm for real-time detection of vital signs of an evacuated person. The proposed solution combines three advanced mechanisms: pulse detection, wristband-to-skin contact verification, and motion detection, enabling reliable and effective monitoring under different conditions. The pulse detection system uses the MAX30102 optical pulse sensor, which detects blood flow changes by analyzing backscattered light. The algorithm is based on statistical signal detection methods, with decision thresholds set according to an assumed miss-detection probability. To do this, the probability density function is estimated using Kernel Density Estimation (KDE). The algorithm employs KDE to estimate the probability density function of the maximum amplitude of the signal spectrum, calculated via FFT. This method ensures the system is resistant to noise and disturbances caused by user movement or varying reflection conditions, maintaining high detection accuracy even in dynamic environments. Additionally, the pulse detection operates effectively at lower LED brightness levels to minimize power consumption, an important factor for wearable devices.

The article also describes a method to verify contact between the wristband and the user’s skin, based on two complementary techniques: analysis of backscattered light brightness and temperature measurement. An IR diode embedded in the MAX30102 sensor emits infrared light, and the detector registers its intensity after reflection from the skin. The average brightness of the reflected light is compared to predefined thresholds with a tolerance margin to account for individual differences like skin pigmentation.

It is notable that an extra thermistor measuring contact point temperature provides additional data to confirm physical contact between the wristband and the body. Combining these techniques reduces false readings caused by improper device placement. In the experimental study, for the scenario “not placed on the hand with movement and without movement” (Table 5), the algorithm achieved a high effectiveness of 100%.

Furthermore, a motion detection method was developed using the MPU-9250 inertial module, which integrates a three-axis accelerometer, gyroscope, and magnetometer. The algorithm analyzes acceleration along the *X*, *Y*, and *Z* axes by calculating the average acceleration and the average absolute deviation from this mean within a time window for each axis. The sum of deviations across all axes creates a motion indicator, which is compared against a threshold determined from empirical data collected during tests in various activity scenarios. This mechanism allows the system to distinguish between rest and motion, essential for accurately interpreting vital signs.

This study presents an analysis of the research results, confirming that the assumed detection error for vital signs did not exceed 20%. The maximum error, resulting from the adopted assumptions and the corresponding parameters of the detection procedure, was 13.89%. This error pertained to the misclassification of a living person at rest.

The error level can be reduced by applying personalized algorithms that enable decision thresholds to be determined based on statistical analyses explicitly conducted for each individual using the wristband.

## 7. Conclusions

This publication introduces an efficient algorithm designed for real-time detection of vital signs in evacuated individuals. Typically, publications focus on detailed monitoring of patients’ vital functions. However, in cases such as evacuation monitoring systems for nursing home residents, complex analysis of pulse or respiratory rate is unnecessary. Instead, quick and effective detection of whether vital signs are present or absent is sufficient.

The paper discusses a device created to provide simple, effective, and energy-efficient vital sign detection. As part of this research, an advanced electronic system was developed to support the evacuation process for individuals with special needs.

In summary, the developed vital signs monitoring system combines accurate pulse detection, skin contact verification through light and temperature analysis, and motion detection using an advanced IMU module. This integration ensures reliable, precise, and adaptive monitoring of the user’s vital functions, accounting for dynamic changes in the wristband’s position. The algorithm’s effectiveness can be enhanced by studying a larger target group. Research involving a broader population will allow for adjustment of the detection threshold, improving correct pulse identification in individuals at rest.

## Figures and Tables

**Table 1:** Comparison of commonly used sensors for monitoring vital signs.

Manufacturer	Model/Series	Characteristics	Application
Analog Devices	MAX30100 [21], MAX30102 [22], MAX86140 [23]	Integrated PPG and SpO_2_ sensor, green/red/IR LEDs, high sensitivity, compact module	Smartwatch, fitness bands, medical monitoring devices
Texas Instruments	AFE4400 [24], AFE4490 [25]	Analog modules for PPG with ultra-low noise and support for multiple wavelengths	Professional wearables, medical devices
Analog Devices	ADPD188GG [26]	Multispectral sensor capable of measuring multiple parameters simultaneously	Advanced health monitoring systems
ams-OSRAM	AS7038 [27]	PPG sensors, highly integrated and low power consumption, electrocardiogram (ECG)	Consumer devices, wearables
ams-OSRAM	SFH7050 [28]	PPG module, electrocardiogram (ECG) and galvanic skin resistance (GSR), up to 8 LED outputs, samples up to 6 photodiode inputs and supports external electrodes, dedicated for wearable devices	Smartwatch, fitness tracker

**Table 2:** Comparison of commonly used IMUs.

Manufacturer	Model/Series	Characteristics	Application
TDK InvenSense Inc.	MPU-9250 [30]	Integrated 9-axis sensor; good accuracy; wide compatibility with microcontrollers; higher power consumption; interference-sensitive magnetometer	Drones, VR, advanced wearables
TDK InvenSense Inc.	MPU-6050 [31]	Cheap, simple to use; extensive documentation; no magnetometer; gyroscope data prone to drift	Education, robots
TDK InvenSense Inc.	ICM-20948 [32]	Improved successor to the MPU-9250; better accuracy; lower noise; more difficult to use; fewer libraries than older models	AR/VR, positioning systems, drones
STMicroelectronics	LSM6DSOX [33]	Low power consumption; advanced features (e.g., Machine Learning Core), higher price; more complex configuration	Wearables, IoT, traffic analytics
STMicroelectronics	LIS3DH [34]	Cheap, simple, very low power consumption; no gyroscope and magnetometer	Smartwatches, sleep trackers, pedometers
Bosch Sensortec	BNO055 [35]	Built-in data fusion system—no need to write an algorithm; higher price; limited refresh rate	VR, motion interfaces, mobile devices
Bosch Sensortec	BMA400 [36]	Ultra-low power consumption; gesture and activity detection; no gyroscope and magnetometer	Fitness bands, IoT, wearables devices

**Table 3:** Comparison of the example values of $P_{MD}$ used to determine the threshold $T_{h}$ with the $P_{MD}$ obtained during testing on the test data.

$\mathrm{Assumed}\;P_{MD}$	$\mathrm{Obtained}\;P_{MD}$	$\mathrm{Value}\;\mathrm{of}\;\mathrm{Threshold}\;T_{h}$
0.005	0.0064	12,344
0.01	0.0106	13,881
0.02	0.0207	17,082
0.05	0.0575	26,257
0.1	0.1061	37,330
0.2	0.2122	61,426

**Table 4:** Percentage of vital signs detected in the four classified cases.

Position of the Monitoring Band	Condition of the Person and the Band	Reading (0) No Pulse and No Movement	Reading (1) No Pulse and Motion Detected	Reading (2) Pulse Detected and No Movement	Reading (3) Pulse Detected and Motion Detected
placed on the wrist	quiescence	13.88%	0%	81.09%	5.02%
not placed on the hand	quiescence	100%	0%	0%	0%
placed on the wrist	while moving	1.68%	4.96%	0.70%	92.66%
not placed on the hand	while moving	0%	100%	0%	0%

**Table 5:** The confusion matrix of vital signs classification.

	Vital Signs Detected	Vital Signs not Detected
Wristband worn on the wrist	89.84%	10.16%
Wristband not worn	0%	100%

## Data Availability

The original contributions presented in this study are included in the article and in publication [39]. Further inquiries can be directed to the corresponding authors.
